# Long Term Use of Personalised Binaural Beats in the Alpha Range: A Pilot Study

**DOI:** 10.3390/bioengineering12121371

**Published:** 2025-12-16

**Authors:** Giacomo Battù, Ludovico Lupo, Silvestro Roatta, Luca Mesin

**Affiliations:** 1 Department of Electronics and Telecommunications, Politecnico di Torino, 10129 Turin, Italy; s319621@studenti.polito.it (G.B.); s317831@studenti.polito.it (L.L.); 2 Department of Neuroscience, University of Torino, 10125 Turin, Italy; silvestro.roatta@unito.it

**Keywords:** Binaural Beats, multivariate analysis, brainwave entrainment

## Abstract

Brainwave entrainment (BWE) through Binaural Beats (BBs) has been proposed as a non-invasive method to modulate cortical activity by enhancing oscillatory power at specific frequencies. Despite growing interest, empirical evidence regarding the efficacy of BBs remains inconsistent. This study aimed to assess long-term effects of BBs stimulation using a personalized protocol. Eleven healthy university students (7 males, 4 females; mean age 24.8 ± 1.6 years) participated in three EEG acquisition sessions over two weeks, each including Baseline, Stimulation, and Post-Stimulation phases. Personalized audio tracks were created based on each participant’s Individual Alpha Frequency (IAF) and applied daily during a 10-day training period. EEG signals were analysed in time and frequency domains using linear and complexity-based metrics. Multivariate processing combining Principal Component Analysis and K-means clustering revealed high classification accuracy distinguishing Baseline from Stimulation (>81%) and Baseline from Post-Stimulation (>89%) phases, with consistent results across sessions and in pooled data. Statistical significance was confirmed via non-parametric permutation testing. Alpha rhythm analysis showed significant frontal effects (F3, F4), including increased spindle incidence, reduced duration, decreased alpha power, and lowered α exponent via Detrended Fluctuation Analysis. Although the dataset was relatively small, these findings suggest that BBs may modulate brain activity, with sustained effects observable post-stimulation, particularly in frontal regions.

## 1. Introduction

Binaural Beats (BBs) are a phantom beat obtained by reproducing two pure tones with slightly different frequencies to the users’ ears [1,2]. The brainwave entrainment hypothesis suggests that when the brain is exposed to external stimulation like the BBs, its electrical activity adjusts to oscillate synchronously with that stimulation [3,4].

The perception of BBs depends on several factors, like the carrier frequency ($f_{0}$), the frequency difference ($f_{BF}$) between two sine waves (at $f_{0}$ and $f_{0}+f_{BF}$, respectively), which also drives the beat frequency ($f_{BF}$), the intensity of the tones, and the presence of noise or music reproduced simultaneously to the pure tones [4]. Concerning the carrier frequency, investigations suggest that below 90 Hz, participants tend to confuse the beat with the generating tones, making the phenomenon difficult to detect as a distinct percept [5]. Furthermore, it is generally accepted that BBs perception declines as the carrier frequency exceeds 1000 Hz [5,6].

Still on BBs perception, it was demonstrated that BBs could be correctly identified with $f_{BF}$ up to 35 Hz, particularly when the carrier frequency was around 400 Hz [6]. The value of $f_{BF}$ also affects the way the sound is perceived. If it is too small, the user perceives a single tone that appears to rotate between ears, also known as a rotating tone. As $f_{BF}$ increases, the listener begins to perceive periodic fluctuations in sound intensity, and if the frequency separation continues to increase, the intensity fluctuations become progressively less distinct. Beyond 20 Hz, the sound becomes rougher and the beat becomes too rapid to be interpreted as an amplitude modulation. Further studies showed that the efficiency of BBs detection depends on the relationship between $f_{0}$ and $f_{BF}$, with optimal perception occurring when $f_{0}$ is between 250 and 500 Hz and $f_{BF}$ is around 10 Hz [7].

Physiological states are associated with a prevalence of specific rhythms in the EEG: e.g., prevalence of alpha activity during relaxation and beta activity during the execution of a cognitive task. For this reason, $f_{BF}$ can be chosen to guide the subject towards a particular mental state [8]. In this sense, given their non-invasiveness, BBs find application in a variety of fields in neuroscience, from working memory enhancement [9,10,11,12] to stress mitigation [2,13,14]. However, despite the encouraging results of some studies, the literature shows mixed results about their efficacy [4]. Furthermore, the consistent variability among the studies in terms of carrier frequency, $f_{BF}$, participants, duration of stimulation and type of stimulation also poses a limitation in terms of comparisons among studies’ results. With particular reference to $f_{BF}$, previous research suggested the possibility of adapting the stimulation frequency to the physiological response of the user, with promising results on stress mitigation on healthy participants [2].

For this reason, in this study, we decided to analyse a personalised stimulation strategy, adopting pure tones, i.e., not corrupted by additive sounds like music, and considering a long exposure to the stimulation. Notably, this is not the first study aiming to address the effect of long exposure to BBs; indeed, a recent study investigated the effect of daily 10-min stimulation at 6 Hz for 1 month, suggesting promising results in terms of cognitive enhancement [15].

In this pilot study, we aim to adopt a similar stimulation condition while incorporating electroencephalogram (EEG) recordings. Unlike previous approaches, however, the $f_{BF}$ is adjusted to each participant’s individual alpha frequency (IAF), thereby tailoring the stimulation to the user’s physiological characteristics. Given the mixed results about BBs efficacy, with this study, we explore if changes in the neural activity manifest before, during and after the subministration of this auditory stimulation.

The rest of this work is structured as follows: Section 2 is dedicated to the description of the participants involved in the study, as well as the techniques adopted for processing the EEG signals. Section 3 presents the results, whereas Section 4 and Section 5 are meant for the discussion and conclusion, respectively.

## 2. Materials and Methods

### 2.1. Participants

The study was conducted on a group of 11 university students (7 males and 4 females) with a mean age of 24.8 ± 1.6 years. The participants recruited in this study reported no hearing impairments or related auditory issues, no history of neurological conditions, concussions, or head trauma, no clinically diagnosed psychological disorders and no prior experience with BBs, in line with the exclusion criteria.

Participants were requested to avoid consuming nicotine, caffeine, memory and concentration supplements, alcohol or narcoleptic drugs at least 3 h before each EEG data collection session, which was repeated multiple times throughout the longitudinal experiment (see Section 2.3). Furthermore, EEG signals were recorded between 10 a.m. and 6 p.m. All the volunteers signed an informed consent before the beginning of the experiment, which was conducted in accordance with the Helsinki declaration. Furthermore, the experimental protocol was approved by the local Ethical Committee (University of Turin—protocol number 0125508).

### 2.2. Instruments

EEGs were recorded with Enobio 8 (Neuroelectrics^®^, Barcelona, Spain), considering 8 channels positioned according to the international 10–10 system, using a neoprene cap. The reference channel (CMS) and the electrode meant for the driven right leg (DRL) were placed on the participant’s earlobe.

In terms of electrode configuration, recordings were obtained from the sites F3, F4, T7, T8, P3, P4, O4, and O5. EEGs were collected with a sampling frequency of 500 Hz.

Regarding BBs, pure tones were generated using a MATLAB^®^ (version R2025a; Natick, MA, USA, The MathWorks Inc.). script, and the resulting 10-min audio tracks were exported in .mp4 format, allowing participants to play them on their smartphones with headphones. For this study, $f_{0}$ was equal to 250 Hz, and the $f_{BF}$ was equal to the IAF of each subject (which was estimated during the first phase of the protocol described below). Specifically, to produce the BBs effect, a sine wave at $f_{0}$ was presented to the left ear, while a sine wave at $f_{0}$ + $f_{BF}$ was delivered to the right ear.

For data processing, the EEGLAB toolbox was employed for the removal of artifacts and noise components from the EEG data.

### 2.3. Experimental Protocol

The experimental protocol consisted of three acquisition phases, each characterized by three steps, during which the participants sat on a chair while keeping their eyes closed using a sleeping mask. Note that EEGs were recorded for the whole duration of the experiment, and the auditory stimulation that the volunteers received was obtained during the first encounter.

**Figure 1:**
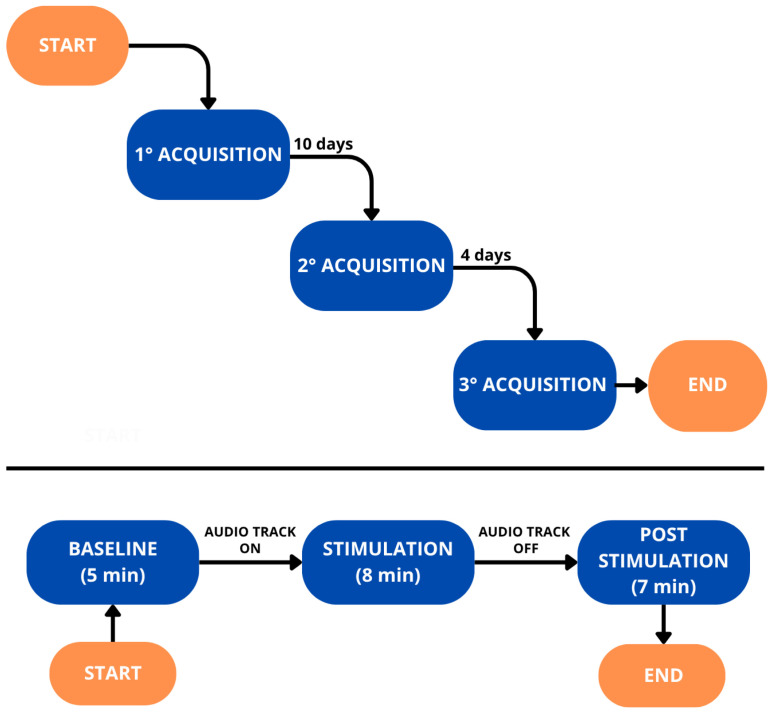
Schematic representation of the experimental protocol. The upper half shows the temporal separation of the acquisition sessions, while the lower half illustrates the individual phases of each session.

The steps of each acquisition phase are described as follows (see Figure 1). First, a resting period (Baseline), lasting approximately 5 min, was observed by the volunteers and during the first ever EEG acquisition of the subject, a personalized audio-track was created considering the individual’ IAF. This parameter was estimated for each channel with the Center of Gravity method, and the average value was used as a representative measure for each participant. Subsequently, the Stimulation phase took place. The personalized audio track was reproduced, and the temporal duration of this phase was 8 min. Lastly, participants observed a new relaxing phase, named in the following as “Post-Stimulation”, aimed at monitoring neural changes in the participants’ EEG resulting from the auditory stimulation. The duration of the last phase was 7 min, and the EEG was collected throughout the duration of the three phases.

After the first data acquisition and audio-track generation, participants were asked to listen to their personalised track once per day for 10 consecutive days, keeping their eyes closed in order to replicate the conditions of the stimulation phase during an acquisition session. Thereafter, a second EEG recording session took place, and from that moment on, participants were requested not to listen to the audio track. Four days later, the last acquisition session was conducted.

Each participant was tested individually and instructed not to discuss any potential effects of the treatment with others. All participants received the stimulation, as no sham or control groups were included due to the limited sample size.

Following data collection for each participant, the primary objective was to assess intra-session differences across phases and to determine whether phase-specific clusters remained separable across multiple recording sessions.

### 2.4. Questionnaires

To monitor the evolution of home-based stimulation, a questionnaire was submitted to patients at the end of the training period, with the aim of assessing the impact of the stimulation on daily life. The questionnaire consisted of eight closed-ended (Yes/No) questions designed to explore the participants’ subjective perception of the stimulation and its effects on overall well-being. The questions were as follows:

- Was the stimulation uncomfortable during the first session?
- Over the days, did your perception of the audio track seem to change?
- During the training period, did you feel relaxed while listening to the audio track?
- During the stimulation sessions, did you feel more relaxed?
- After the stimulation sessions, did you feel more relaxed?
- During the experimental period, did your sleep feel more regular and restful?
- After completing the experiment, would you have liked to continue listening to the audio track on your own?

### 2.5. EEG Analysis

EEG signal analysis was conducted using a multivariate approach, combining the principal component analysis (PCA) [16,17] with K-means clustering [18,19] to assess if it was possible to separate data relative to the different conditions: Baseline, Stimulation and Post-Stimulation.

**Figure 2:**
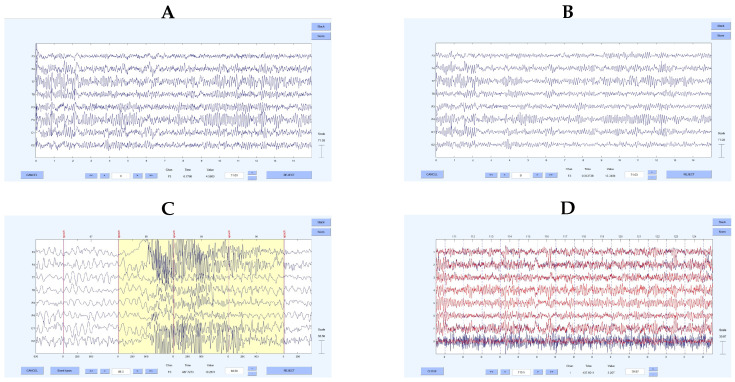
Screenshots from the EEGLAB toolbox, illustrating the different preprocessing steps applied to the EEG signals. (**A**) Raw EEG signals. (**B**) Filtered and re-referenced EEG signals. (**C**) Example of EEG epochs containing artifacts (i.e., yellow shading portion) that were removed after visual inspection. (**D**) Noise and physiological artifact removal using ICA decomposition, in blue the original traces while in red the cleaned ones.

To obtain a signal that accurately reflects the brain’s electro-cortical activity, the raw EEG traces underwent a thorough cleaning process, as shown in Figure 2: the first pre-processing steps consisted of a change in the EEG signals’ reference, through the average referencing technique [20]; then signals were band-pass filtered using a 5th-order, zero-lag, anti-causal Butterworth filter, with a high-pass cut-off frequency of 0.5 Hz and a low-pass cut-off frequency of 45 Hz, to preserve the EEG frequency components of interest. Epochs with abnormal activity were removed through visual inspection using the EEGLAB toolbox. Finally, the Independent Components Analysis (ICA), based on the Extended Infomax Algorithm [21,22] and implemented in the MATLAB’s EEGLAB Toolbox, was performed to identify and separate signal sources corresponding to physiological/non-physiological artefacts superimposed on the EEG recordings, with the aim of obtaining a signal representing only cortical activity.

EEGs were then characterised using linear and non-linear techniques, exploring both the time and frequency domains. The features extracted from the linear analysis included band power across standard frequency bands: delta (0.5–4 Hz), theta (4–8 Hz), alpha (8–13 Hz), beta (13–30 Hz), and gamma (30–45 Hz). For each frequency band previously mentioned, both the absolute band power and the ratio of the band power to the total power of the signal (0.5–45 Hz) were computed. Furthermore, the power within a sub-range of the alpha band was computed, defined as a ±1 Hz window centred on the IAF, along with its contribution to the total alpha power. The exploration of the linear features was completed by assessing the alpha asymmetry index (AAI) and the Hjorth parameters (Activity, Mobility, and Complexity).

Regarding non-linear metrics, Higuchi and Katz fractal dimensions and the Spectral Entropy (SE) were considered, as well as connectivity metrics like the phase locking value (PLV). All the described metrics were assessed in 15-s segments, and all the features, of the same acquisition session, were standardized with a Z-score approach.

After performing PCA, Principal Components (PCs) were ranked according to the explained variance of the projected data. K-means clustering was then applied to the space of the selected PCs to assess whether the resulting clusters corresponded to the experimental conditions (Baseline, Stimulation and Post-Stimulation) [23]. K-means was initialised with k=2, corresponding to the number of phases analysed in each comparison, focusing first on Baseline vs. Stimulation and then on Baseline vs. Post-Stimulation. The initial centroids were set based on the mean characteristics of the two distributions to facilitate convergence. The K-means analysis was performed at two levels: acquisition level (observations from each acquisition session for all subjects were analysed independently) and subject level (observations from all three acquisitions for each subject were combined, projected into a common PCs space, and then analysed).

**Figure 3:**
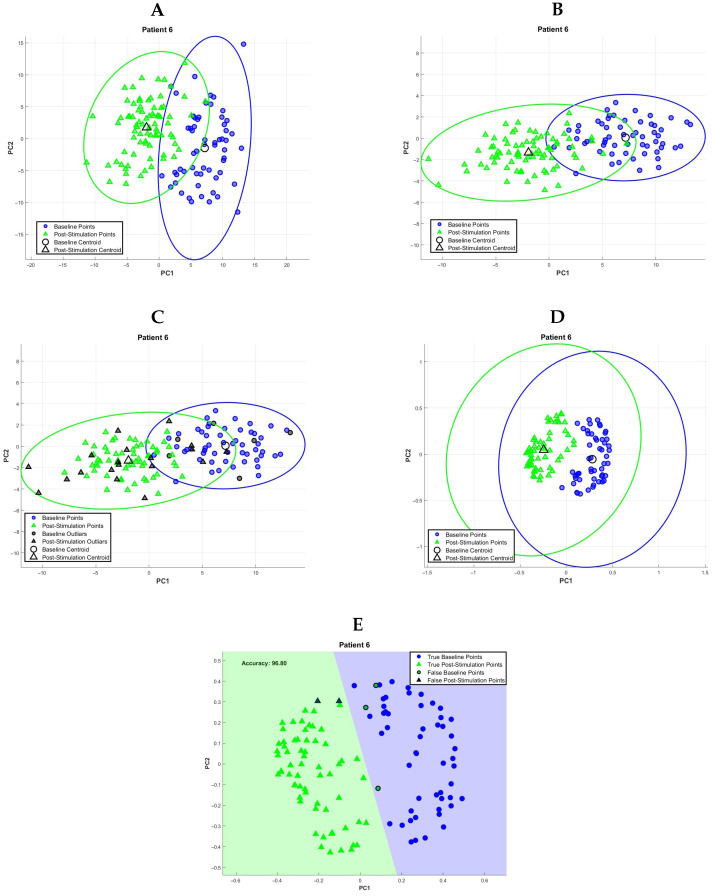
K-means clustering pipeline. An example of the classification between Baseline vs. Post-Stimulation is shown, where each element of the distributions corresponds to a single EEG epoch. (**A**) Projection of data on PCs space relative to a single subject. (**B**) Projection of data on PCs space after principal components selection. (**C**) Projection of data on PCs space with the outlier highlighted. (**D**) Laplacian kernel applied to the projection of data on the PCs space. (**E**) K-means clustering algorithm output.

Several optimisation steps were introduced to enhance the separation between conditions, as shown in Figure 3. First, feature selection was performed using the Fisher Score [24], with a threshold of 0.8 applied to retain only the most informative features. Next, the PCs obtained by the application of PCA on the most informative features went through a selection process by the Random Forest algorithm [25], which assigns an importance score to each component based on its ability to distinguish between classes (Baseline, Stimulation and Post-Stimulation); only components ranked in the top 50% were retained for further analysis. To reduce the influence of outliers, observations were further filtered based on the Mahalanobis distance [26], keeping only those within the 90th percentile of their class distribution, effectively removing data points that significantly deviated from the average behaviour of each class. Finally, a Laplacian kernel [27] was applied in the principal component space, introducing a non-linear element that improves the model’s capacity to capture complex class boundaries. Given the various processing steps involved in the analysis, and considering the reduced size of the sample, a nonparametric permutation test was carried out to evaluate the presence of overfitting, and the results are presented in the Results Section (see Section 3).

### 2.6. Alpha Rhythm Analysis

Once the results from the multivariate analysis of the EEG signal were obtained, it was possible to confirm the presence of distinct mental states during the different phases of the experimental design. However, this analysis did not provide detailed information about the underlying neural dynamics. To gain deeper insight, an Alpha Rhythm Analysis was conducted to extract and characterise the alpha spindles within the frequency band where the stimulation frequency lies.

In particular, to assess whether BBs interfere with the spontaneous modulation of alpha rhythm amplitude, a detailed morphological analysis of alpha spindles, i.e., fluctuations within the alpha rhythm exhibiting a typical ‘waxing and waning’ amplitude pattern [28,29], was carried out, complemented by a complexity analysis of the alpha rhythm envelope.

The signal x(t), representing the cleaned EEG trace, was first filtered using a 4th-order zero-lag anti-causal Butterworth band-pass filter between 8 and 12 Hz, yielding the narrow-band process $x^{\alpha }\left(t\right)$, representing the alpha activity. The following steps for computing the envelope and extracting alpha spindles were carried out according to the procedure described in the work of Sano et al. [28]: the envelope of the analytical version of $x^{\alpha }\left(t\right)$ was then computed, followed by a 200 ms moving average filtering to retain the slower fluctuations in amplitude while attenuating high frequency ones. The envelope’s first derivative was used for the alpha spindles extraction to detect local peaks and valleys through zero-crossing analysis. Alpha spindles were then defined as the portions of the alpha rhythm between two valid valleys containing at least one valid peak, ensuring the expected waxing-waning shape.

The obtained alpha spindles were morphologically characterised in duration, mean amplitude and incidence rate (number of spindles per second) [30]. In addition, the α-exponent was computed from the alpha rhythm envelope using the Detrend Fluctuation Analysis (DFA) [31], providing an index of long-range temporal correlation within the signal [32].

### 2.7. Statistical Analysis

Statistical analyses of the collected data were performed using MATLAB^®^ (MathWorks, Inc., Natick, MA, USA, R2024a). To assess the absence of over-fitting in the results due to the multiple steps of the K-means clustering pipeline, a permutation test was implemented in which the labels were randomized prior to running the pipeline. This procedure allowed us to test if the clustering outcome was not driven by the specific analysis steps performed. The entire analysis, including clustering, was then repeated 50 times with different random label assignments and a *p*-value was computed empirically as the proportion of permutation runs in which the clustering accuracy obtained with randomized labels exceeded that of the original data. Passing on to the results due to the alpha rhythm analysis, to evaluate the effects of multiple treatments, the two-way Scheirer-Ray-Hare test [33] was applied, considering treatment and subject as factors, without including interaction terms, to account for repeated measurements. Pairwise comparisons were conducted using the Wilcoxon signed-rank test. For the alpha rhythm, Cohen’s d was also calculated to quantify the effect size of the observed differences, providing a measure of the magnitude of the effect independent of sample size. A significance level of α=0.05 was set for all analyses.

## 3. Results

This section presents the results obtained from the behavioural questionnaire and the EEG multivariate analysis. The following paragraphs describe the main findings regarding participants’ subjective responses and the classification performance of the EEG data. Regarding the responses obtained from the questionnaire, 63.6% of participants reported that the stimulation was not perceived as unpleasant during the first session. However, 81.8% indicated that the stimulation became more pleasant over the following days, including all participants who initially found it unpleasant, and 54.5% stated that their perception of the stimulation changed over time. With respect to the perceived effects, 81.8% of the participants reported feeling more relaxed during the training period, with the same proportion (81.8%) stating that they experienced a sense of relaxation already during the stimulation itself, which persisted afterwards [34,35]. Conversely, 81.8% of the respondents did not notice any beneficial effects on sleep. Finally, 54.5% of the participants declared that they would have continued self-performing the stimulation even after the experimental phase had ended.

**Figure 4:**
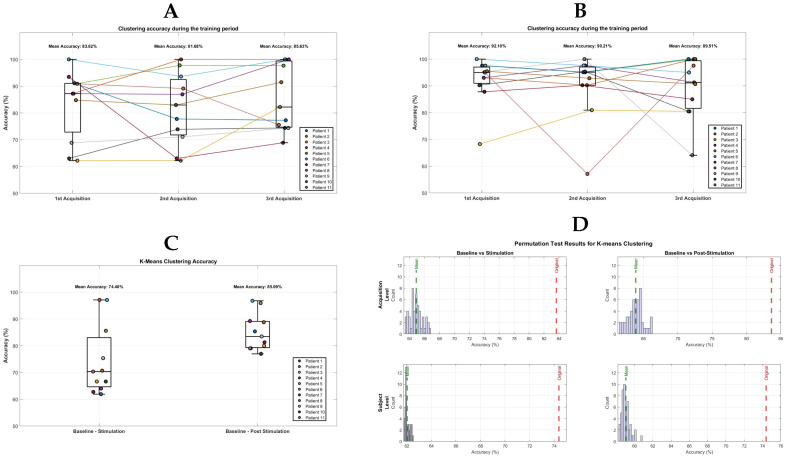
(**A**) Baseline vs. Stimulation clustering performance on the acquisition level. Each boxplot refers to the K-means accuracy distribution obtained on each acquisition. The round markers represent the accuracy of each subject. (**B**) Baseline vs. Post-Stimulation clustering performance on the acquisition level. Each boxplot refers to the K-means accuracy distribution obtained on each acquisition. The round markers represent the accuracy of each subject. (**C**) The boxplot on the left shows the distribution of classification accuracy for the comparison between Baseline and Stimulation, while the boxplot on the right refers to the comparison between Baseline and Post-Stimulation. The results are based on the aggregated analysis of observations from all three acquisitions corresponding to the subject-level analysis. (**D**) Permutation test results for K-means clustering of EEG-based accuracy measures. Each subplot shows the distribution of clustering accuracies obtained from 50 random label permutations (blue histogram) compared with the original observed accuracy (red dashed line, labelled “Original”) and the mean of the permutation distribution (green dashed line, labelled “Mean”). The top row represents the acquisition-level analysis, contrasting Baseline vs. Stimulation (left) and Baseline vs. Post-Stimulation (right), while the bottom row represents the subject-level analysis for the same comparisons.

Turning to the results obtained from the EEG multivariate analysis, to address the first question concerning the possibility of correctly clustering epochs belonging to different phases within the same acquisition session, Figure 4A illustrates the temporal evolution of clustering accuracy for the Baseline vs. Stimulation comparison across the three acquisitions. The observations from each class were correctly classified with an average accuracy of 83.62% for the first acquisition, 81.68% for the second, and 85.63% for the third. As shown in Figure 4B, the classification accuracy for the Baseline vs. Post-Stimulation comparison was higher than that for the Baseline vs. Stimulation comparison across all three acquisitions, with mean accuracy values of 92.10%, 90.21%, and 89.51%, respectively.

A qualitative inspection of the accuracy in discriminating the different conditions suggests a subject-dependent consistency: participants for whom the discrimination accuracy was initially lower (or higher) tended to exhibit similar levels in subsequent sessions. This observation may reflect inter-individual differences in the strength of participants’ responses to BB stimulation.

The second aim was to evaluate whether it was still possible to correctly cluster all epochs belonging to the same phase across all acquisition sessions of the same subject. Figure 4C illustrates the K-means clustering performance in distinguishing between different experimental conditions within a projection space characterised by a higher noise level. Specifically, the average accuracy obtained for the Stimulation vs. Baseline comparison was 74.40% (on the left of the image), whereas the Post-Stimulation vs. Baseline comparison yielded a mean accuracy of 85.09% (on the right of the image).

After obtaining the clustering results, a non-parametric permutation test was performed to assess the significance of the observed clustering patterns. For each test, the labels were randomised prior to applying the K-means clustering algorithm, and this randomization procedure was repeated 50 times to evaluate the potential presence of over-fitting. The analysis was conducted at both the acquisition level and the subject level. The results, as seen in Figure 4D, indicated that the clustering performance obtained on the original data was significantly higher than the distribution of performance values obtained from the permuted datasets. This finding held consistently when comparing Baseline vs. Stimulation, as well as Baseline vs. Post-Stimulation.

**Figure 5:**
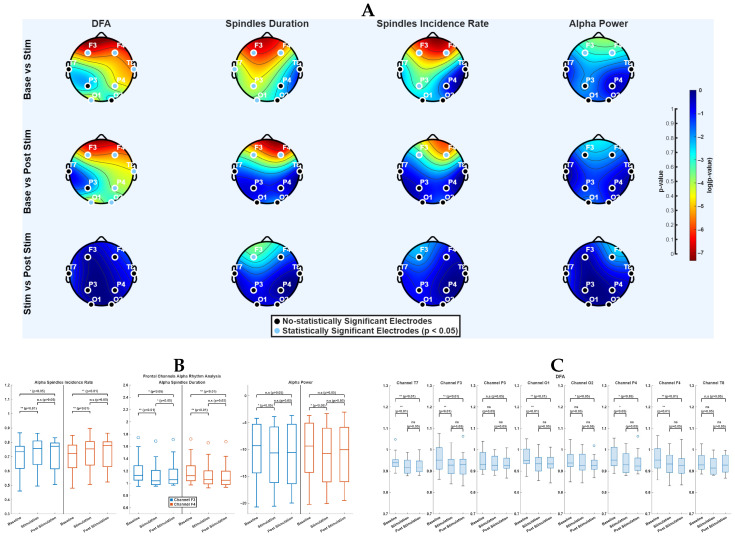
(**A**) Each row corresponds to a different comparison between experimental phases, while columns represent distinct features extracted from the alpha rhythm. In each topomap, the dots are the electrode positions; light blue dots highlight the channels where a statistically significant difference was found. The background colourmap encodes the $\log_{10}$ *p* values obtained from the statistical test. (**B**) Box plots illustrate alpha rhythm metrics across the three experimental phases (Baseline, Stimulation, Post-Stimulation) for frontal EEG channels F3 (blue) and F4 (orange). On the left there is the Alpha Spindles Incidence Rate, in the middle the Alpha Spindles Duration, while on the right Alpha Power. Statistical comparison between different phases is indicated above each plot (n.s. = not significant). (**C**) Box plots illustrate Detrended Fluctuation Analysis (DFA) α exponent across all EEG channels during the three experimental phases (Baseline, Stimulation, Post-Stimulation). Statistical comparison between different phases is indicated above each plot (n.s. = not significant).

To gain a deeper insight into the effect of BBs on the brain rhythms at the stimulation frequency, the analysis focused on the characterisation of alpha spindles. Figure 5A illustrates the graphical results of repeated measures statistical tests conducted on the features used to characterise the alpha rhythm across different experimental conditions. During the stimulation phase, the α-exponent showed significant changes in most channels (with the exception of P3, T7, and O1), with moderate effect sizes (e.g., d=−0.43 for F3 and F4). Regarding spindle duration, significant differences were observed predominantly in the left hemisphere, including channel F3, where a small effect was detected (d=−0.27), while other affected channels showed smaller effects (e.g., O2: d=−0.16). The spindle incidence rate also differed from Baseline, particularly in the frontal region and at channel P3, with small effect sizes (F3: d=0.30, F4: d=0.22, P3: d=0.19). In addition, significant changes in alpha band power were detected at channels F3 and F4, both showing small effect sizes (F3: d=−0.14, F4: d=−0.14). During the post-stimulation phase, the spatial pattern of significant channels for the α-exponent remained largely consistent with the Stimulation phase, except for channel O2. As shown in Figure 5C, the α-exponent tended to decrease both during and after BBs stimulation, indicating an increase in signal complexity, with moderate effect sizes (e.g., F3: d=−0.46, F4: d=−0.37). For both spindle duration and spindle incidence rate, significant changes were confined to the frontal region, again with small effects (e.g., F3 duration: d=−0.18; F3 incidence: d=0.20). In contrast, no channels outside the frontal area exhibited statistically significant differences in alpha band power, and the associated effect sizes remained small (e.g., F4: d=−0.11). More specifically, the frontal region appears to be strongly affected by the alpha rhythm modulation induced by the BBs.This modulation is reflected in morphological changes in alpha spindle behaviour, characterised by an increased Incidence Rate and a decreased duration, in channels F3 and F4, as seen in Figure 5B. Finally, no significant differences were observed between the Stimulation and Post-Stimulation conditions for any of the features extracted from alpha spindles, with all effect sizes remaining very small (|d|<0.10).

## 4. Discussion

The present pilot study explored the potential effects of continuous and pure BBs stimulation on cortical signals. Both subjective/qualitative data and quantitative/EEG-based information were recorded.

Overall, the responses to the questionnaire indicated that the stimulation was well tolerated, with most participants not perceiving it as unpleasant during the initial session and the majority reporting that it became progressively more pleasant over time, even among those who initially found it uncomfortable. This progressive change in perception may reflect the adaptability of participants to auditory stimulation and suggests that repeated exposure could enhance its acceptability. In terms of perceived effects, a large proportion of participants reported feelings of relaxation both during stimulation and throughout the training period, and these effects appeared to persist beyond the sessions, consistent with previous findings suggesting that BBs can modulate emotional and physiological states [34,35]. By contrast, most of the respondents did not notice improvements in sleep quality, indicating that potential sleep-related benefits may require longer or more structured protocols. Interestingly, a majority of participants expressed willingness to continue the practice independently, suggesting that BBs may serve as a valuable tool for supporting or enhancing meditative states.

Complementing these subjective findings, the clustering analysis applied on EEGs, although based on a limited dataset, suggests that cortical activity may differ across experimental conditions, i.e., during and after stimulation, compared to Baseline. The need for multivariate analysis to discriminate these conditions further indicates that BBs stimulation may not reliably modulate individual EEG features, but could induce small, distributed changes detectable only through integration of multiple sources of information. These results also point to a considerable degree of variability (both between subjects and between sessions) in terms of the features that exhibit the most significant differences between conditions. The fact that observations from the Stimulation and Post-stimulation phases remain distinguishable from the Baseline even in aggregate analysis, despite the increased complexity and variability inherent to such a comparison, suggests some robustness and repeatability of effects induced by BBs. Another surprising finding is that the separation between experimental conditions appeared even more pronounced when the Post-stimulation phase was evaluated relative to the Baseline, potentially exceeding the separation observed between the Stimulation and Baseline phases. This may indicate that the effects of BBs consolidate after the stimulation audio track is turned off, although it is also possible that cognitive processing of the auditory stimulation during the Stimulation phase attenuates its effects.

The results of the alpha rhythm analysis suggest that stimulating at the IAF may induce morphological modulation of the cerebral rhythm, corresponding to the target frequency. Specifically, the frontal cerebral region, especially channels F3 and F4, exhibited the most noticeable changes relative to Baseline, which remain statistically significant even during the Post-stimulation phase. The observed increase in the incidence rate of alpha spindles, combined with a reduction in their average duration, may reflect partial synchronisation of the alpha rhythm in the frontal channels with the BBs. These beats manifest as short-duration amplitude modulations occurring at a high temporal frequency. Furthermore, exclusively on the frontal channels, a significant reduction in alpha band power was observed. These findings are consistent with previous results, which showed that continuous, unmasked stimulation using pure beats primarily affects the frontal region of the cerebral cortex [36], and that stimulation within the alpha band may lead to a reduction in the overall power of the alpha rhythm [37].

Finally, the α exponent derived from the DFA appeared to be a robust metric for detecting the effects of BBs on the alpha rhythm. The results showed a statistically significant reduction in this parameter in nearly all brain regions, both during and after stimulation, suggesting that BBs may modulate the temporal dynamics of alpha rhythm fluctuations. Notably, healthy subjects have been observed to exhibit a reduction in the α exponent during focused attention meditation tasks [38], which may imply that stimulation through BBs could induce a state of concentration.

By contrast, no clear evidence emerged regarding the effectiveness of the training period in enhancing the stimulation effects, either in PCA combined with K-means clustering or in the alpha rhythm analysis.

### Limitations

Despite the promising results obtained, this study has some limitations that should be addressed in future research. One of the main limitations concerns the relatively small sample size (11 participants), which may reduce the statistical power of the analyses, limit the generalisation of the results and prevent meaningful comparisons with a control group, such as a group that receives no stimulation or one that receives stimulation at a fixed frequency allowing to better interpret BBs effects. Moreover, the study exclusively involved university students. On one hand, this choice reduced sample heterogeneity and helped limit potential confounding factors; on the other hand, our dataset could not fully represent the cognitive profiles of other age groups or demographics, thereby highlighting the need for further studies.

From a technical analysis perspective, the lack of clear and widely accepted markers of cerebral responses to BBs makes it difficult to evaluate the stimulation’s long-term effects and demonstrate its effectiveness. Furthermore, only the alpha band was analysed as this corresponds to the stimulation frequency used in the experiment. Future work could benefit from exploring whether similar effects occur in other frequency bands, which might provide a more comprehensive understanding of BBs modulation. The experimental design could also be improved. Increasing the number of recording sessions per subject, ideally through daily monitoring, and increasing both the training period duration and the phase in which home BBs stimulation is suspended would support more robust longitudinal analysis.

Another limitation relates to instrumentation: employing a high-density acquisition system would allow for more detailed topographical and functional connectivity analysis, and better utilisation of the ICA algorithm for identifying with greater accuracy which brain areas are most involved during the stimulation. It is also important to highlight the lack of standardisation in the audio playback system. Participants used their own personal devices, including earphones and smartphones, which inevitably introduced variability in sound quality and volume intensity, potentially affecting the stimulation effects. However, the longitudinal design of the study, requiring daily BBs exposure, made it necessary to adopt a flexible protocol that could be easily integrated into the participants’ daily routines.

In addition, another crucial aspect concerns the lack of shared guidelines in the scientific community regarding the optimal parameters (e.g., carrier and stimulation frequency, lateralisation) for targeting specific cognitive and psychological effects [39,40]. Another field requiring further investigation concerns the mode of BBs stimulation. It is still unclear whether stimulation should be continuous and prolonged or whether it is more effective when delivered in blocks or through bursts [2].

Given these open issues, future studies should focus on a systematic analysis of BBs to clarify the mechanisms of action and identify the optimal conditions for effective stimulation. In light of the results obtained in this work, it may be promising to move toward personalised stimulation protocols, and implementing a real-time adjustment of the parameters could further enhance the effectiveness of the BBs.

## 5. Conclusions

This work contributed to clarify key aspects of BBs stimulation, particularly when the stimulation frequency is calibrated to each subject’s IAF. Results highlighted an overlooked aspect in studies involving BBs: the subjectivity of individual response to stimulation. Moreover, the in-depth analysis of the alpha rhythm allowed for a more accurate exploration of the brain entrainment mechanisms associated with BBs stimulation.

## Data Availability

The datasets presented in this article are not readily available because the data are part of an ongoing study. Requests to access the datasets should be directed to the corresponding author.
